# Prevalence of chronic low back pain: systematic review

**DOI:** 10.1590/S0034-8910.2015049005874

**Published:** 2015-10-05

**Authors:** Rodrigo Dalke Meucci, Anaclaudia Gastal Fassa, Neice Muller Xavier Faria

**Affiliations:** I Universidade Federal do Rio GrandeFaculdade de MedicinaUniversidade Federal do Rio GrandeRio GrandeRSBrasil Divisão de População e Saúde. Faculdade de Medicina. Universidade Federal do Rio Grande. Rio Grande, RS, Brasil; II Universidade Federal de PelotasUniversidade Federal de PelotasPelotasRSBrasil Programa de Pós-Graduação em Epidemiologia. Universidade Federal de Pelotas. Pelotas, RS, Brasil; III Prefeitura Municipal de Bento GonçalvesPrefeitura Municipal de Bento GonçalvesBento GonçalvesRSBrasilPrefeitura Municipal de Bento Gonçalves. Bento Gonçalves, RS, Brasil

**Keywords:** Low Back Pain, epidemiology, Pain Measurement, Prevalence, Review

## Abstract

**OBJECTIVE:**

To estimate worldwide prevalence of chronic low back pain according to age and sex.

**METHODS:**

We consulted Medline (PubMed), LILACS and EMBASE electronic databases. The search strategy used the following descriptors and combinations: back pain, prevalence, musculoskeletal diseases, chronic musculoskeletal pain, rheumatic, low back pain, musculoskeletal disorders and chronic low back pain. We selected cross-sectional population-based or cohort studies that assessed chronic low back pain as an outcome. We also assessed the quality of the selected studies as well as the chronic low back pain prevalence according to age and sex.

**RESULTS:**

The review included 28 studies. Based on our qualitative evaluation, around one third of the studies had low scores, mainly due to high non-response rates. Chronic low back pain prevalence was 4.2% in individuals aged between 24 and 39 years old and 19.6% in those aged between 20 and 59. Of nine studies with individuals aged 18 and above, six reported chronic low back pain between 3.9% and 10.2% and three, prevalence between 13.1% and 20.3%. In the Brazilian older population, chronic low back pain prevalence was 25.4%.

**CONCLUSIONS:**

Chronic low back pain prevalence increases linearly from the third decade of life on, until the 60 years of age, being more prevalent in women. Methodological approaches aiming to reduce high heterogeneity in case definitions of chronic low back pain are essential to consistency and comparative analysis between studies. A standard chronic low back pain definition should include the precise description of the anatomical area, pain duration and limitation level.

## Abstract (pt)

**OBJETIVO:**

Estimar a prevalência mundial de dor lombar crônica, segundo idade e sexo.

**MÉTODOS:**

Foram consultadas as bases de dados eletrônicas Medline (PubMed), Lilacs e Embase. A estratégia de busca utilizou os seguintes descritores: *back pain, prevalence*_*,*_* musculoskeletal diseases*_*,*_* chronic musculoskeletal pain*_*,*_* rheumatic*_*,*_* low back pain*_*,*_* musculoskeletal disorders *e* chronic low back pain*. Foram selecionados os estudos de base populacional de delineamento transversal ou coortes que avaliaram dor lombar crônica como desfecho. A qualidade dos estudos selecionados foi avaliada, assim como a prevalência de dor lombar crônica, segundo idade e sexo.

**RESULTADOS:**

Foram incluídos 28 estudos nesta revisão. De acordo com a avaliação qualitativa, cerca de um terço dos estudos tiveram pontuação baixa, principalmente em decorrência das altas taxas de não respondentes. A prevalência de dor lombar crônica foi de 4,2% em indivíduos com idade entre 24 e 39 anos e 19,6% entre aqueles de 20 a 59 anos. Dentre nove estudos com indivíduos com 18 anos ou mais, em seis a prevalência de dor lombar crônica variou entre 3,9% a 10,2%, e nos outros três estudos a prevalência foi entre 13,1% e 20,3%. Entre idosos brasileiros, a prevalência de dor lombar crônica foi de 25,4%.

**CONCLUSÕES:**

A prevalência de dor lombar crônica aumenta linearmente a partir da terceira década de vida até os 60 anos de idade, sendo mais prevalente nas mulheres. Questões metodológicas visando a reduzir a alta heterogeneidade nas definições de casos nos estudos sobre dor lombar crônica são fundamentais para permitir análises comparativas e de consistência entre diferentes estudos. A definição de dor lombar crônica deve incluir a descrição precisa da área anatômica, período de duração da dor e nível de limitação.

## INTRODUCTION

Low back pain is a common condition affecting many individuals at some point in their lives.^4^ The estimation is that between 5.0% and 10.0% of cases will develop chronic low back pain (CLBP), which is responsible for high treatment costs, sick leave, and individual suffering,^26^^-^^28^ in addition to being one of the main reasons for people to seek health care services.^13^^,^^28^ Although CLBP is highly disabling, information about its prevalence and associated factors are scattered in the literature. Most results are presented in a secondary way in studies evaluating several musculoskeletal outcomes simultaneously. Moreover, we found great variability among studies as to the characterization of chronic and low back pain. A systematic review of the global prevalence of low back pain included a summary prevalence of chronic low back pain.^21^ However, the prevalence estimates found by the authors were based on studies with great variability concerning anatomical characterization of the low back region. Thus, the included studies have definitions according to which back and/or neck pain were considered low back pain.^21^ This lack of standardization disregard specificities of the cervical, thoracic and lumbar spine as well as the attempts in the literature to standardize low back pain studies.^11^

The objective of this review was to estimate the worldwide chronic low back pain prevalence according to age and sex.

## METHODS

We consulted electronic databases without any restrictions regarding language or year of publication, and the final database search took place on June 8, 2014. We searched terms as words to broad the number of references retrieved.

The search strategy varied according to the database, as follows:

Medline: back pain [Mesh] AND prevalence [Mesh], chronic musculoskeletal pain prevalence, rheumatic low back pain, musculoskeletal disorders low back pain prevalence, chronic low back pain AND prevalence;

LILACS: back pain AND prevalence, chronic musculoskeletal pain prevalence, rheumatic low back pain, musculoskeletal disorders low back pain prevalence, chronic low back pain AND prevalence;

EMBASE: back pain AND prevalence, chronic musculoskeletal pain prevalence, rheumatic low back pain, musculoskeletal disorders low back pain prevalence, “chronic low back pain” AND “prevalence”.

All references retrieved from the databases were exported to EndNote^®^. To identify duplicated studies, we used the EndNote^®^ “find duplicates” tool configured to compare titles and authors from the retrieved references, and manually excluded duplicates not identified by the program.

In the review, we excluded publications with titles that enabled the identification of studies conducted with specific populations such as students, occupational groups or individuals with specific illnesses as well as literature reviews. In the following stage, we read the abstracts. Those that enabled the identification of literature reviews or studies assessing musculoskeletal outcomes other than chronic low back pain and studies using convenience samples were also excluded.

After the abstracts, the studies selected were read and excluded if they assessed occupational groups, used convenience samples, or if they lack definition on the anatomical location of low back pain or the period of time determining pain as being chronic. Studies assessing chronic low back pain in individuals with low back pain, which provide insufficient information to calculate the prevalence of this outcome in the entire sample, were also excluded.

The searches focused on population-based or cohort studies evaluating CLBP prevalence. Only studies with a clear definition of low back pain and time criteria for pain chronicity were selected.

We identified the following characteristics of the selected studies: country, response rate, number of individuals evaluated/interviewed, age group, low back pain definition, use of human body drawings, and chronic pain definition. CLBP prevalence was then extracted and the confidence interval was calculated for those studies without information about it.

The studies were evaluated according to a quality tool adapted from Hoy et al,^21^ which included eight items: sample representativeness, sample size estimates, census or random sampling process, non-respondent bias probability, primary data collection, validated questionnaire instrument, standardized data collection, and human body drawings (Table 1). A score index was built whereby a weighting of 0.2 was attributed to sample representativeness, census or random sample, and non-respondent bias probability. A weighting of 0.08 was attributed to the remaining five items, thus enabling a maximum score of 1. More weighting was attributed to those characteristics with greater potential of causing bias in chronic low back pain prevalence estimates.

**Table 1:** Chronic low back pain according to population-based studies.

Author (year)	Country	Design	Response rate	N	Male		Female		Age or age group	Definition of chronic pain	Prevalence	95%CI
			%		n	%	n	%			%	
Hoddevik et al^20^ (1999)	Norway	CS	63.4	67,338	31,846	47.3	35,492	52.7	40-42	> 3 months	2.0	1.9;2.1
Shiri et al^38^ (2008)	Finland	CS	76.0	2,575	1,185	46.0	1,390	54.0	24-39	Continuous pain in the last year	4.2	3.4;5.0
Picavet et al^36^ (2000)	Netherlands	CS	50.0	22,415	10,132	45.2	12,283	54.8	20-59	> 3 months	19.1	18.6;19.6
Palmer et al^34^ (2005)	England	CS	53.0	2,632	Not reported	Not reported	Not reported	Not reported	25-64	> 6 months	11.0	9.8;12.2
Hillman et al^19^ (1996)	England	CS	72.0	3,184	1,437	45.1	1,747	54.9	25-64	> 3 months	10.2	9.1;11.3
Alkherayf et al^1^ (2009)	Canada	CS	78.9	73,507	35,242	47.9	38,265	52.1	20-59	Continuous pain > 6 months	19.6	19.3;19.9
Picavet et al^37^ (2003)	Netherlands	CS	50.0	3,664	1,640	44.8	2,024	55.2	≥ 25	> 3 months	21.2	19.9;22.5
Heuch et al^18^ (2010a)	Norway	CS	69.0	63,968	30,102	47.1	33,866	52.9	≥ 20	> 3 months	23.6	23.3;23.9
Bjorck-Van Dijken et al^6^ (2008)	Sweden	CS	69.3	5,798	Not reported	Not reported	Not reported	Not reported	25-79	> 6 months	16.4	15.5;17.4
Johannes et al^24^ (2010)	USA	CS	75.7	27,035	10,357	38.3	16,678	61.7	≥ 18	> 6 months	8.1	7.5;8.7
Carey et al^8^ (1995)	USA	CS	79.0	8,067	Not reported	Not reported	Not reported	Not reported	≥ 21	> 3 months/or 24 episodes of pain in the last year	3.9	3.5;4.3
Freburger et al^14^ (2009)	USA	CS	86.0	9,924	Not reported	Not reported	Not reported	Not reported	≥ 21	> 3 months/or 24 episodes of pain in the last year	10.2	9.6;10.8
Meucci et al^29^ (2013)	Brazil (Pelotas)	CS	89.6	2,732	1,151	42.1	1,581	57.9	≥ 20	≥ 7 weeks in the last 3 months	9.6	8.3;10.8
Andersson^5^ (1994)	Sweden	CS	90.0	1,609	817	50.8	792	49.2	25-74	> 3 months	23.3	21.2;25.4
Silva et al^39^ (2004)	Brazil (Pelotas)	CS	94.4	3,182	1,374	43.2	1,808	56.8	≥ 20	≥ 7 weeks in the last 3 months	4.2	3.5;5.0
Almeida et al^2^ (2008)	Brazil (Salvador)	CS	97.1	2,281	1,016	44.5	1,265	55.5	≥ 20	Continuous pain > 6 months	14.7	13.3;16.2
Dellaroza et al^9^ (2013)	Brazil (Sao Paulo)	CS	89.9	1,271	513	40.4	758	59.6	≥ 60	Continuous pain > 6 months	25.4	23.0;27.8
Omokhodion^31^ (2002)	Nigeria	CS	100	900	450	50.0	450	50.0	20-85	> 3 months	7.0	5.3;8.7
Brattberg et al^7^ (1989)	Sweden	CS	82.0	857	391	47.3	436	52.7	18-84	> 6 months	20.3	17.6;23.0
Altinel et al^3^ (2008)	Turkey	CS	100	2,035	841	41.3	1,194	58.7	≥ 19	Continuous pain	13.1	11.6;14.6
Park et al^35^ (1993)	USA	CS	87.0	44,233	18,562	42.0	25,671	58.0	≥ 18	> 3 months	6.7	6.4;7.0
Fujii et al^15^ (2012)	Japan	CS	Not reported	52,650	26,779	50.9	25,871	49.1	20-79	4^th^ degree low back pain lasting > 3 months at some time in life	3.9	3.7;4.1
Jacobsson et al^22^ (1989)	Sweden	CS	49.4	445	230	51.7	215	48.3	50-69	Pain > 6 weeks Rheumatologist’s diagnosis	6.3	4.0;8.6
Liao et al^26^ (2009)	China	CS	88.7	10,921	5,687	52.1	5,234	47.9	≥ 16	> 3 months	1.0	0.8;1.2
Jimenez-Sanchez et al^23^ (2012)	Spain	CS	Not reported	12,190	5,742	47.1	6,448	52.9	≥ 16	> 3 months	11.1	10.5;11.7
Hagen et al^16^ (2011)	Norway	C	HUNT 2: 53.0	49,483	Not reported	Not reported	Not reported	Not reported	≥ 20	> 3 months	–	–
			HUNT 3: 54.0	50,839							HUNT 2: 22.7	22.4;23.0
			Wave II:90 WI	1,671							HUNT 3: 23.4	23.0;23.9
Van Oostrom et al^32^ (2011)	Netherlands	C	Baseline: 62.0	12,405	2,686	47.1	3,020	52.9	26-65	> 3 months or “pain always present” Definitions changed during follow-ups T1-T2	Follow-up I: 17.4%	
			Follow-up I: 79.0	6,118							Follow-up II: 17.4%	
			Follow-up II: 75.0	4,917							Follow-up III: 19.9%	
			Follow-up III: 78.0	4,520							Incidence: No/No/No: 62.4% No/Yes/Yes or No/No/Yes: 10.8%	
			For analyses with data from all follow-ups	5,706							Recurrence/Persistence: Yes/Yes/No or Yes/No/No: 10.3% Yes/No/Yes or No/Yes/No: 10.9% Yes/Yes/Yes: 5.6%	
Waxman et al^40^ (2000)	England	C	Baseline: 76.0	3,184	–		–		25-64	> 3 months	Baseline: 6.3% Follow-up: 11.1%	
			Follow up: 70.0	1,455	615		840					

CS: cross-sectional; C: cohort; HUNT: Nord-Trøndelag Health Study

We reported this systematic review according to the PRISMA Statement.^30^

## RESULTS

**Figure 1:**
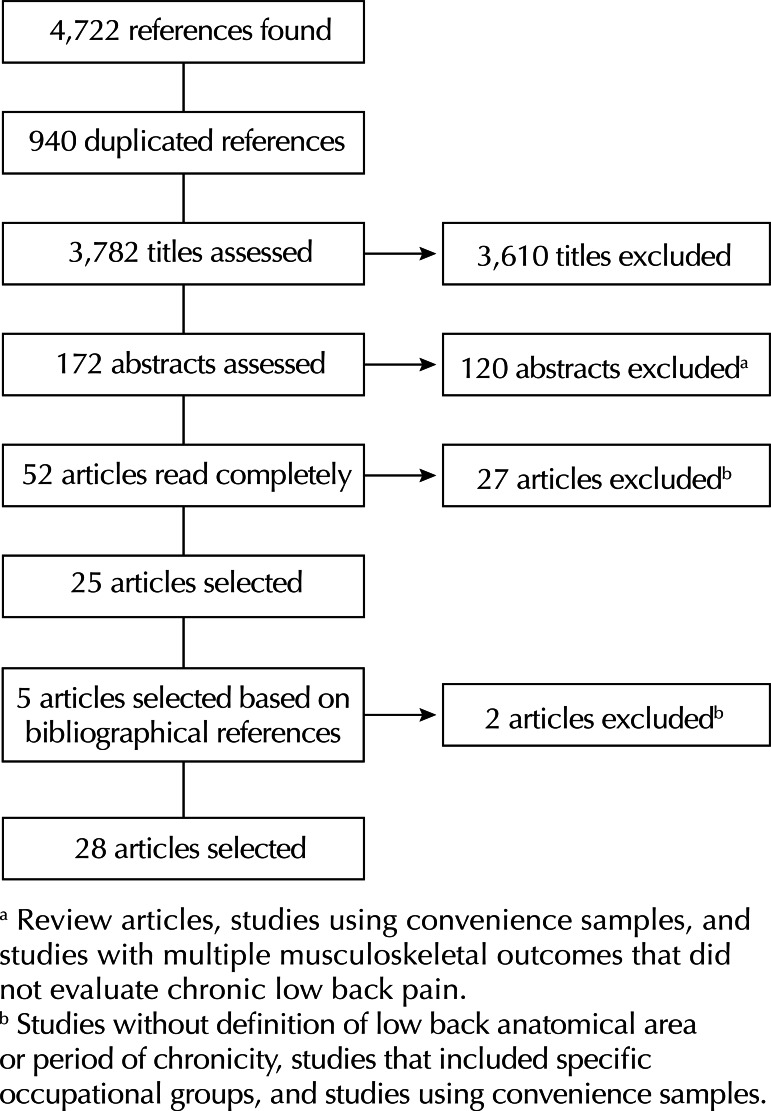
Selection process for studies of chronic low back pain prevalence.

We found twenty-eight studies that fulfilled the inclusion criteria, which were thus included in this review (Figure 1). Of the twenty-five original population-based cross-sectional studies, 13 were European,^3^^,^^5^^-^^7^^,^^18^^-^^20^^,^^22^^,^^23^^,^^31^^,^^33^^-^^35^ five were North American (USA and Canada),^1^^,^^8^^,^^14^^,^^24^^,^^32^ four were South American (Brazil),^2^^,^^9^^,^^29^^,^^37^ two were Asian (Japan and China)^15^^,^^26^ and one was African (Nigeria)^31^ (Table 1). The response rate was greater than 75.0% in fifteen studies. Two articles did not report the response rate (Table 1).

Regarding studies using a population-based cohort design, a Norwegian study performed a census of the population aged over 20 in a given province and did not report the proportion of males and females.^16^ The other studies used random sampling of individuals of both sexes aged between 20 and 65.^38^^,^^39^ The follow-up rates of the cohort studies varied between 53.0% and 79.0% (Table 1).

Thirteen of the population-based cross-sectional studies defined chronic pain as a period of continuous pain lasting more than three months; seven used a “over six months” criterion, two used continuous pain, two others used pain lasting for more than seven weeks, and one used pain lasting for more than six weeks. All three population-based cohort studies used the same criterion (pain lasting more than three months).

Regarding the qualitative analysis of the reviewed papers, all studies achieved scores in their description of a census or random sampling process, primary data collection, and standardized data collection; 27 studies had representative samples of the target population; 19 studies had small non-respondent bias probability; only four articles described the sample size estimates; three papers evaluated the study questionnaire reliability; and 10 studies used human body drawings to locate low back pain (Table 2).

**Table 2:** Qualitative evaluation of the assessed studies.

Study	Score weight								Total score
	0.2	0.08	0.2	0.2	0.08	0.08	0.08	0.08	

	Was the sampling frame a true or close representation of the target population?	Was the sample size estimated?	Was some form of random selection used to select the sample, OR, was a census undertaken?	Was the likelihood of non-response bias minimal?	Were data collected directly from the subjects (as opposed to a proxy)?	Had the study instrument that measured the parameter of interest (e.g., CLBP prevalence) been tested for reliability and validity (if necessary)?	Was data collection standard ized?	Was a human body drawing used?	
Hoddevik et al^20^ (1999)	Yes	No	Yes	No	Yes	No	Yes	No	0.56
Shiri et al^38^ (2008)	Yes	No	Yes	Yes	Yes	No	Yes	Yes	0.84
Picavet et al^36^ (2000)	Yes	No	Yes	No	Yes	No	Yes	Yes	0.64
Palmer et al^34^ (2005)	Yes	No	Yes	No	Yes	No	Yes	No	0.56
Hillman et al^19^ (1996)	Yes	No	Yes	No	Yes	Yes	Yes	Yes	0.72
Alkherayf et al^1^ (2009)	Yes	No	Yes	Yes	Yes	No	Yes	No	0.76
Picavet et al^37^ (2003)	Yes	No	Yes	No	Yes	No	Yes	Yes	0.64
Heuch et al^18^ (2010a)	Yes	No	Yes	No	Yes	No	Yes	No	0.56
Bjorck-Van Dijken et al^6^ (2008)	Yes	No	Yes	No	Yes	No	Yes	No	0.56
Johannes et al^24^ (2010)	Yes	No	Yes	No	Yes	No	Yes	No	0.76
Carey et al^8^ (1995)	Yes	No	Yes	Yes	Yes	No	Yes	No	0.76
Freburger et al^14^ (2009)	Yes	No	Yes	Yes	Yes	No	Yes	No	0.76
Meucci et al^29^ (2013)	Yes	Yes	Yes	Yes	Yes	No	Yes	Yes	0.92
Andersson^5^ (1994)	Yes	No	Yes	Yes	Yes	Yes	Yes	Yes	0.92
Silva et al^39^ (2004)	Yes	Yes	Yes	Yes	Yes	No	Yes	Yes	0.92
Almeida et al^2^ (2008)	Yes	Yes	Yes	Yes	Yes	No	Yes	No	0.84
Dellaroza et al^9^ (2013)	Yes	No	Yes	Yes	Yes	No	Yes	No	0.76
Omokhodion^31^ (2002)	Yes	No	Yes	Yes	Yes	No	Yes	Yes	084
Brattberg et al^7^ (1989)	Yes	No	Yes	Yes	Yes	No	Yes	No	0.76
Altinel et al^3^ (2008)	Yes	Yes	Yes	Yes	Yes	No	Yes	No	0.84
Park et al^35^ (1993)	Yes	No	Yes	Yes	Yes	No	Yes	No	0.76
Fujii et al^15^ (2012)	Yes	No	Yes	Yes	Yes	No	Yes	Yes	0.84
Jacobsson et al^22^ (1989)	Yes	No	Yes	No	Yes	No	Yes	No	0.56
Liao et al^26^ (2009)	Yes	No	Yes	Yes	Yes	No	Yes	No	0.76
Jimenez-Sanchez et al^23^ (2012)	Yes	No	Yes	Yes	Yes	No	Yes	No	0.76
Hagen et al^16^ (2011)	Yes	No	Yes	Yes	Yes	Yes	Yes	No	0.64
Van Oostrom et al^32^ (2011)	Yes	No	Yes	Yes	Yes	No	Yes	No	0.76
Waxman et al^40^ (2000)	Yes	No	Yes	Yes	Yes	No	Yes	Yes	0.84

CLBP: chronic low back pain

According to the score index, nine studies scored between 0.56 and 0.64. The main reason for the low scores found by these studies were their high non-response rates. Eleven studies scored between 0.72 and 0.76. Most of these did not obtain scores for instrument validation, use of human body drawings, and sample size calculation. Eight studies scored between 0.84 and 0.92, and the items that resulted in these high scores were “use of medical manikin” or “human body drawing”, and “sample size calculation” (Table 2).

Considering only cross-sectional population-based studies with response rates above 75.0%, CLBP prevalence was 4.2% in individuals aged 24 to 39^38^ years and 19.6% in those aged 20 to 59.^1^ In six out of nine studies^2^^,^^3^^,^^7^^,^^8^^,^^14^^,^^24^^,^^29^^,^^31^^,^^39^ with individuals aged 18, 19, 20, 21 years or above, CLBP varied between 3.9% and 10.2%.^8^^,^^14^^,^^24^^,^^29^^,^^31^^,^^39^ Three reported higher prevalence rates (13,1%, 14.7%, and 20.3%).^2^^,^^3^^,^^7^ CLBP prevalence was 23.3% in individuals aged 25 to 74^5^ (Table 1) and 25.4% among older adults (≥ 60 years old).^9^ We found no difference in relation to CLBP prevalence at different periods of the year or in different places.

**Figure 2:**
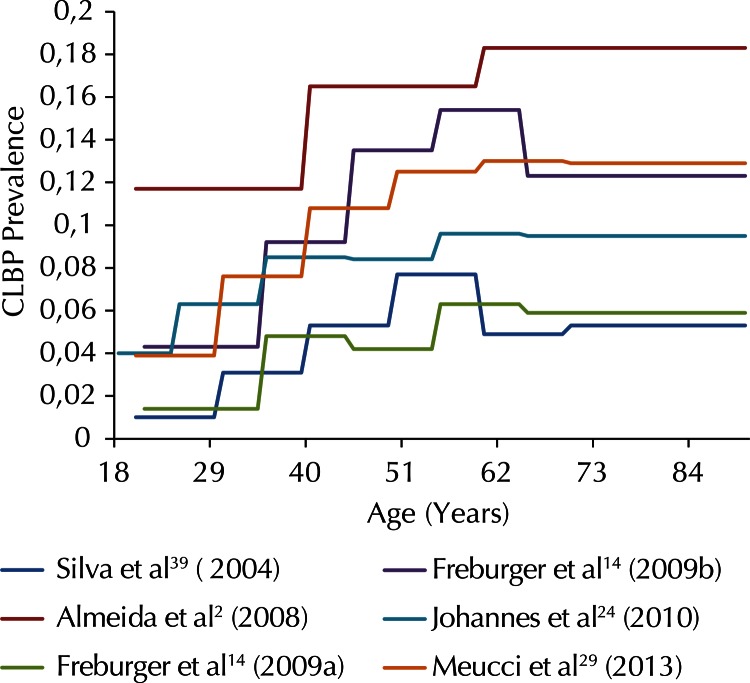
Chronic low back pain prevalence (CLBP) according to age (six estimates).

Five studies with high response rates presented CLBP prevalence according to specific age groups.^2^^,^^14^^,^^24^^,^^29^^,^^39^Figure 2 shows that CLBP prevalence rates are lower in younger individuals (aged 20 to 30 years), increasing from the third decade of life on, reaching the highest proportions between 50 and 60 years of age, and stabilizing in the seventh decade of life.

Two studies (Figure 2) showed that CLBP occurrence has doubled in recent years in North Carolina and in Pelotas in all age groups analysed.^14^^,^^29^

**Figure 3:**
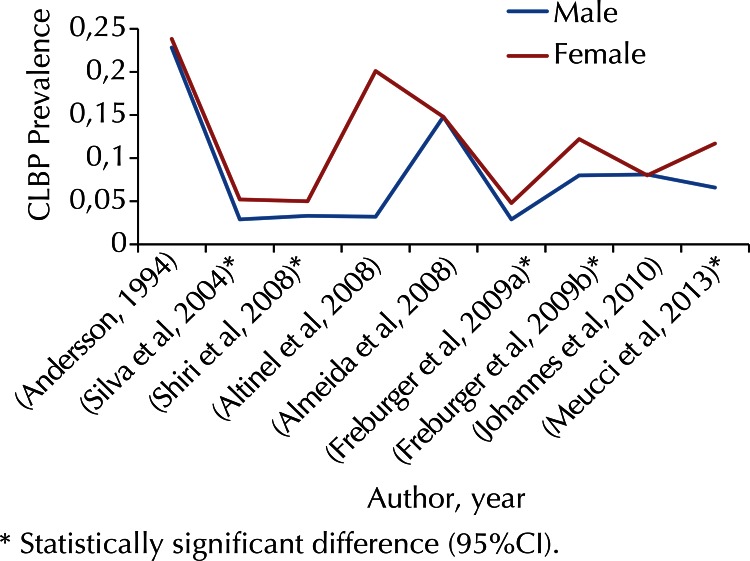
Chronic low back pain (CLBP) according to sex (nine estimates).

In five^2^^,^^14^^,^^24^^,^^29^^,^^39^ of nine^2^^,^^3^^,^^7^^,^^8^^,^^14^^,^^24^^,^^29^^,^^31^^,^^39^ studies with individuals (or older than) 18, 19, 20, or 21 years old and response rates above 75.0%, CLBP prevalence was around 50.0% higher in women than in men (Figure 3).

Only eight studies^1^^,^^2^^,^^14^^,^^15^^,^^23^^,^^29^^,^^32^^,^^39^ evaluated CLBP prevalence using other independent variables. One study showed that CLBP prevalence is higher in white and black non-Hispanic individuals in relation to Hispanic individuals.^14^ Four studies showed that individuals with less schooling have more CLBP than those with more schooling.^15^^,^^23^^,^^29^^,^^39^ Two studies found that individuals of lower economic status had higher CLBP prevalence than those of higher economic status.^29^^,^^39^ Six studies assessed CLBP prevalence using smoking as a variable. In all six studies, smokers had more CLBP than non-smokers.^1^^,^^2^^,^^15^^,^^29^^,^^32^^,^^39^ Three studies^29^^,^^32^^,^^39^ found that obese individuals have more CLBP than eutrophic individuals (Table 3).

**Table 3:** Chronic low back pain according to other variables in population-based studies, except age and sex.

Author (year)	Variable	Prevalence			
		%	95%CI	%	95%CI
Alkherayf et al^1 ^(2009)	Smoking status	Daily smokers (present or former): 23.3 Occasional smokers (present or former): 17.2 Non-smokers: 15.7 Analysis stratified by smoking status: CLBP prevalence was higher in daily smokers (present or former) in comparison to occasional smokers (present or former) and non-smokers in all variables assessed: sex, age, BMI, education and occupational status			

Freburger et al^14^ (2009)	Race/Ethnicity	1992		2006	
		Non-Hispanic white: 4.1	3.5;4.7	Non-Hispanic white: 10.5	9.4;11.5
		Non-Hispanic black: 3.0	2.0;4.0	Non-Hispanic black: 9.8	8.2;11.4
		Other:4.1	1.4;6.8	Hispanic: 6.3	3.8;8.9
				Other: 9.1	6.2;12.0

Meucci et al^29^ (2013) & Silva et al^39^ (2004)	Education (years)	2002		2010	
		0: 6.9	6.0;7.8	0: 14.3	9.7;18.9
		1-4: 6.3	5.5;7.2	1-4: 13.0	10.2;15.7
		5-8: 4.4	3.7;5.2	5-8: 9.7	7.5;11.9
		9-11: 2.7	2.2;3.3	9-11: 8.1	5.9;10.2
		≥ 12: 2.0	1.5;2.6	≥ 12: 6.8	4.7;8.8
	Economic status	A or B: 2.8	2.3;3.4	A or B: 7.8	5.0;10.5
		C: 4.6	3.9;5.4	C: 9.0	7.4;10.5
		D or E: 4.6	3.9;5.4	D or E: 11.3	9.0;13.6
	Smoking	Never: 3.2	2.6;3.9	Never: 8.0	6.6;9.4
		Former smoker: 5.0	4.3;5.8	Former smoker: 11.3	8.5;14.1
		Smoker: 5.5	4.7;6.3	Smoker: 11.5	9.2;13.9
	BMI (kg/m^2^)	≤ 19.9: 2.7	2.1;3.3	≤ 19.9: 4.3	0.5;8.0
		20-24.9: 3.4	2.8;4.1	20-24.9: 8.0	6.1;9.8
		25-29.9: 4.1	3.4;4.9	25-29.9: 8.4	6.5;10.2
		≥ 30.0: 6.2	5.7;7.1	≥ 30.0: 14.2	11.5;16.9

Almeida et al^2^ (2008)	Smoking	Never: 12.2			
		Former smoker: 19.7			
		Smoker: 17.6			
	Marital status	Married or partner: 15.9			
		Single: 9.5			
		Widow or divorced: 20.6			

Fujii^15^ (2012)	Smoking	No CLBP		CLBP	
		Ever smoked: 52.4		Ever smoked: 42.6	
	Education	College: 49.4		College: 40.8	

Jimenez-Sanchez et al^23^ (2012)	Education	Male		Female	
		No studies: 9.7	6.9;13.5	No studies: 20.1	16.7;24.0
		Primary: 9.9	8.7;11.2	Primary: 17.1	15.7;18.6
		Secondary:6.6	5.4;7.9	Secondary: 10.7	9.3;12.3
	Marital status	Single: 4.3	3.4;5.4	Single: 7.7	6.5;9.1
		Married: 9.5	8.6;10.6	Married: 15.5	14.3;16.8
		Divorced or widowed: 10.5	7.2;15.1	Divorced or widowed: 20.4	18.0;23.0

Van Oostrom**et al^32^ (2011)		Analysis stratified by 3 patterns of low back pain: never long-standing LBP; persistent LBP over 10 years; varying LBP. Individuals with persistent LBP were less educated, have less paid job, were more obese, and predominantly smokers.			

CS: cross-sectional; C: cohort; LBP: low back pain; BMI: Body Mass Index; CLBP: Chronic Low Back Pain.

According to the population-based cohort studies, CLBP prevalence was of 6.3% in England and 23.0% in Norway.^16^^,^^32^^,^^40^ CLBP incidence in at least one follow-up session was 10.8%, whereas persistence in all three follow-up sessions was 5.6% (Table 1).^32^

## DISCUSSION

Almost half the studies included in this systematic review had a response rate lower than 75.0%. The criteria for chronic low back pain case definition are heterogeneous. The most common criterion was continuous pain for a period equal to or greater than three months. Based on our qualitative evaluation, around one third of the studies obtained low scores, mainly due to high non-response rates. CLBP prevalence varied according to the age ranges in the studies and was around three to four times higher in individuals aged over 50 compared to those aged 18 to 30. Females, people of lower economic status, those with less schooling, and smokers had higher CLBP prevalence compared to males, people with higher economic status, those with more schooling, and non-smokers, respectively.

In relation to the quality of the studies, the instrument used showed that the main characteristic that reduced their score was the high rate of non-respondents. This limitation makes clear the challenge to reduce the proportion of non-respondents in population-based studies, especially in countries where postal surveys are used. The instrument used included eight evaluation questions contemplating most items applicable to observational studies on the checklist proposed by Downs and Black,^12^ mainly concerning sample representativeness. In this review, we attributed more weight to these items.

Two studies indicated that CLBP prevalence doubled over time.^14^^,^^29^ This might reflect important changes in lifestyle and in the world of work. The intensive use of computers at work and at home as well as other technologies has increased sedentariness – a risk factor for chronic and acute low back pain due to muscle weakness.^17^^,^^25^ Obesity is also related to lifestyle and is a known risk factor for CLBP as it promotes overloading of the articular structures of lumbosacral spine, which become predisposed to degeneration.^29^

The increase in CLBP prevalence among individuals aged 30 to 60 may also be related to occupational and domestic exposures that overload the low back along with the degenerative articular process shown after 30 years of age. Although CLBP stabilizes or reduces from the seventh decade of life on, its prevalence remains high when compared to younger individuals (aged 20-30). This reduction among older people may be due to reduced exposure to occupational and everyday activities that increase the risk for CLBP.^2^^,^^14^^,^^24^^,^^29^^,^^39^ The literature also suggests that older adults are more resilient to pain due to factors related to ageing, such as cognitive impairment and decreased pain perception.^21^

The mechanism whereby females have consistently higher CLPB prevalence is partially known.^2^^,^^3^^,^^5^^,^^14^^,^^24^^,^^29^^,^^38^^,^^39^ This might be related to women’s exposure to musculoskeletal loads due to pregnancy, child care, and double workday (domestic tasks plus paid work). Furthermore, physiological characteristics such as less muscle and bone mass as well as psychological factors may contribute to higher CLBP prevalence among them.^21^

Higher CLBP prevalence in individuals with less income and less schooling may be related to inferior living and working conditions, which can lead them to jobs that have greater risk to the lumbar spine.^29^ Regarding the higher proportion of CLBP among smokers, this is caused by the systemic effects of nicotine on the joints of the spine, accelerating the joint degeneration process, and increasing the potential of transmission of pain impulses in the central nervous system.^29^^,^^39^ According to the literature, overweight or obese individuals are subject to greater loads on the lumbar spine, thus favoring the development of chronic pain in this region.^29^^,^^39^

Hoy et al^21^ made a valuable contribution to low back pain studies and estimated a summary prevalence of CLBP of 20.1% (SD = 9.8). However, these results should be critically evaluated given that this prevalence estimation included inaccurate outcome definitions such as back and neck as synonyms for low back.^21^ Our systematic review used a stricter definition of CLBP for low back location. Moreover, having CLBP as a primary focus of interest allowed more in-depth discussion on its specificities, which are usually dispersed among time periods of varying durations estimating how recently pain occurred.

Although this systematic review only included studies with a precise definition of low back pain regarding its anatomical location, heterogeneity in chronic pain definition may have influenced the prevalence rates reported, and this is therefore a limitation to our study. Similarly, since CLBP is frequently a secondary outcome, little information are available about its prevalence to other covariables and this is a significant gap in knowledge regarding CLBP.

Moreover, the lack of standardized methods between studies about the subject hinders the evaluation of occurrence measurements and CLBP associated factors in observational studies, as well as the evaluation of the treatment efficacy for this problem. Therefore, methodological approaches aiming to reduce high heterogeneity are key to provide consistency and comparative analysis between different studies, systematic reviews, and meta-analysis. A standard CLBP definition should include the anatomical area of reference, period of pain evaluation, limitation level, and proper differentiation between acute and CLBP. These recommendations are in keeping with the recent National Institute of Health (NIH) Pain Consortium Task Force on research standards for CLBP, which defined this outcome as a back pain problem that has persisted for at least three months and has resulted in pain on at least half the days in the past six months. NIH suggested a minimum data set for evaluating CLBP, which includes a human body drawing showing the lumbar spine, as well as studying limitations in everyday activities arising from CLBP.^10^

Moreover, CLBP studies need some improvement in developing countries and other regions, given that the large concentration of studies in European countries shows higher CLBP prevalence in older populations, mainly in Caucasian individuals with better living conditions.
